# Comparative Genomics and Transcriptome Profiling in Primary Aldosteronism

**DOI:** 10.3390/ijms19041124

**Published:** 2018-04-09

**Authors:** Elke Tatjana Aristizabal Prada, Isabella Castellano, Eva Sušnik, Yuhong Yang, Lucie S. Meyer, Martina Tetti, Felix Beuschlein, Martin Reincke, Tracy A. Williams

**Affiliations:** 1 Medizinische Klinik und Poliklinik IV, Klinikum der Universität, Ludwig-Maximilians-Universität München, 80336 Munich, Germany; elke_tatjana_aristizabal.prada@med.uni-muenchen.de (E.T.A.P.); Eva.Susnik@med.uni-muenchen.de (E.S.); Yuhong.Yang@med.uni-muenchen.de (Y.Y.); Lucie.Meyer@med.uni-muenchen.de (L.S.M.); Felix.Beuschlein@med.uni-muenchen.de (F.B.); Martin.Reincke@med.uni-muenchen.de (M.R.); 2 Division of Pathology, Department of Medical Sciences, University of Torino, 10124 Torino, Italy; isabella.castellano@unito.it; 3 Division of Internal Medicine and Hypertension, Department of Medical Sciences, University of Torino, 10126 Torino, Italy; marty_queen@alice.it; 4 Klinik für Endokrinologie, Diabetologie und Klinische Ernährung, UniversitätsSpital Zürich, CH-8091 Zurich, Switzerland

**Keywords:** primary aldosteronism, aldosterone, aldosterone-producing adenoma, transcriptome profiing

## Abstract

Primary aldosteronism is the most common form of endocrine hypertension with a prevalence of 6% in the general population with hypertension. The genetic basis of the four familial forms of primary aldosteronism (familial hyperaldosteronism FH types I–IV) and the majority of sporadic unilateral aldosterone-producing adenomas has now been resolved. Familial forms of hyperaldosteronism are, however, rare. The sporadic forms of the disease prevail and these are usually caused by either a unilateral aldosterone-producing adenoma or bilateral adrenal hyperplasia. Aldosterone-producing adenomas frequently carry a causative somatic mutation in either of a number of genes with the *KCNJ5* gene, encoding an inwardly rectifying potassium channel, a recurrent target harboring mutations at a prevalence of more than 40% worldwide. Other than genetic variations, gene expression profiling of aldosterone-producing adenomas has shed light on the genes and intracellular signalling pathways that may play a role in the pathogenesis and pathophysiology of these tumors.

## 1. Introduction

Primary aldosteronism (PA) is the most common potentially curable form of hypertension with a prevalence of 5–10% in patients with hypertension [1,2,3] and is characterized by the excessive production of aldosterone. Aldosterone excess has detrimental effects independent of blood pressure control as demonstrated by the increased risk of cardiovascular and cerebrovascular events and target organ damage in patients with PA relative to matched patients with essential hypertension. PA is mainly caused by either an aldosterone-producing adenoma (APA) or bilateral adrenal hyperplasia (BAH). The diagnosis of an APA is appealing because hypertension can be cured or markedly improved by unilateral adrenalectomy and resolve the aldosterone excess in the majority of cases [4]. In contrast, patients with BAH are usually treated with a mineralocorticoid receptor antagonist but plasma renin levels should be monitored as a therapeutic response (as well as blood pressure) because suppressed renin, independent of blood pressure control, is associated with an increased risk of cardiometabolic events and death relative to patients with essential hypertension [5]. A landmark in PA research was the discovery of germline and somatic mutations that drive the aldosterone overproduction in patients with PA, discoveries that were made possible by the application of next-generation sequencing [6,7,8,9,10,11,12]. The genetic basis of four familial forms of PA has now been described as well as somatic mutations in APAs and these are discussed in more detail below.

Herein, we outline novel contributions and major discoveries that have been made in the field of PA research over the last few years. We describe the genetic basis of familial forms of hyperaldosteronism and the identification of somatic mutations that lead to excess aldosterone production. Differential gene expression profiles between APAs and reference tissues are highlighted, as well as key signalling pathways and molecular mechanisms that may drive cell proliferation and constitutive aldosterone production in APAs. Additionally, the possible influence of genetics and genomics on surgical outcome and the potential application of next-generation sequencing methods and transcriptome profiling as possible prognostic tools are described.

## 2. Synthesis of Aldosterone

**Figure 1:**
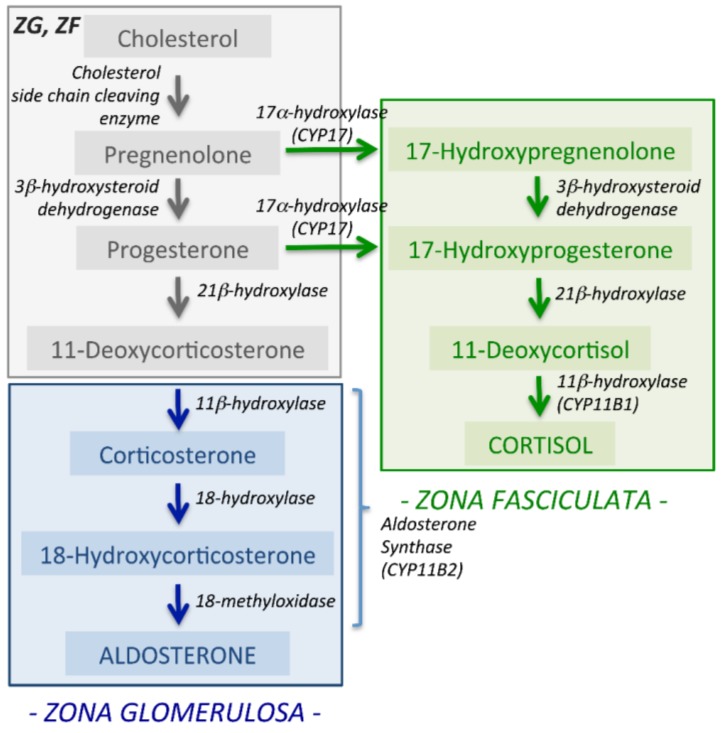
Aldosterone synthesis in the adrenal cortex. Aldosterone is synthesized in the *zona glomerulosa* (ZG) distinct from the synthesis of cortisol in the *zona fasciculata* (ZF). Aldosterone synthase encoded by *CYP11B2* performs all three enzymatic steps in the conversion of deoxycorticosterone to aldosterone.

The primary function of aldosterone is to maintain fluid and electrolyte balance for the control of blood pressure. The main physiological regulators of aldosterone synthesis are angiotensin II, potassium and adrenocorticotropic hormone. Aldosterone synthesis is restricted to the *zona glomerulosa* (ZG), the outer layer of the adrenal cortex (Figure 1) where aldosterone synthase converts deoxycorticosterone to aldosterone by three successive steps of 11β-hydroxylation, 18-hydroxylation and 18-oxidation by a single enzyme, aldosterone synthase (encoded by *CYP11B2*). *CYP11B2* displays a high level of intron and exon sequence homology to the *CYP11B1* gene localized in the *zona fasciculata* (ZF) that encodes 11β-hydroxylase that catalyses the final step in the conversion of 11-deoxycortisol to cortisol (Figure 1). Angiotensin II and potassium regulate aldosterone production via Ca^2+^ signalling which also plays a key role in the aldosterone excess in PA due to the somatic and germline mutations in ion channels and transporters.

## 3. Familial Forms of Hyperaldosteronism

There are currently 4 recognised forms of familial hyperaldosteronism (FH types I–IV) and the genetic basis of each type is summarized in Table 1.

### 3.1. Familial Hyperaldosteronism Type I

Familial hyperaldosteronism type I (FH type I or GRA, glucocorticoid remediable aldosteronism) is caused by a hybrid *CYP11B1/CYP11B2* gene inherited as an autosomal dominant trait. The hybrid gene results from an asymmetrical crossing over between the highly homologous *CYP11B1* (encoding 11β-hydroxylase) and *CYP11B2* (encoding aldosterone synthase) genes and comprises 5′ sequences of *CYP11B1* (including the promoter region) and 3′ sequences of *CYP11B2* (including the coding region of aldosterone synthase). Thus, in FH type I, aldosterone synthase is ectopically expressed in the ZF under the control of adrenocorticotropic hormone (ACTH) rather than restricted to the ZG under the control of angiotensin II [13,14].

### 3.2. Familial Hyperaldosteronism Type II

Familial hyperaldosteronism type II (FH type II) was first described by Stowasser et al. [15] in a kindred with an autosomal dominant form of PA. Other kindreds were subsequently described and a linkage with a locus on chromosome 7p22 was reported in some but not all families but sequencing the entire linked locus did not identify the genetic cause [16]. In 2018, Scholl et al. [17] identified the genetic variant responsible in the original kindred with FH type II described by Michael Stowasser as a heterozygous variant of the *CLCN2* gene that caused early-onset primary aldosteronism and hypertension often with hypokalaemia. *CLCN2* encodes CIC-2, a homodimer voltage-gated chloride channel expressed in the adrenal gland predominantly in the ZG [17]. In the original family with FH type II, eight individuals were carriers of the *CLCN2* mutation (resulting in the CIC-2 p.Arg172Gln substitution) and of these, seven tested positive with a screening test for primary aldosteronism (elevated aldosterone-to-renin ratio). One carrier for the CIC-2 p.Arg172Gln variant had a normal aldosterone-to-renin ratio, and therefore did not have primary aldosteronism, indicating an incomplete penetrance of the allele. Scholl et al. found the p.Arg172Gln substitution in an additional kindred and two further cases of p.Arg172Gln mutations (1 occurring de novo) in 2 unrelated patients with early-onset PA [17] as well as other *CLCN2* variants encoding 4 different mutations in CIC-2 (a de novo p.Met22Lys mutation, p.Tyr26Asn, p.Ser865Arg and p.Lys362del). At the same time, Fernandes-Rosa et al., reported a de novo heterozygous p.Gly24Asp mutation in the CIC-2 chloride channel associated with PA [18]. Electrophysiological recordings showed that the mutated CIC-2 channels display modified gating resulting in increased chloride efflux compared with wild-type CIC-2 channels. The increased chloride efflux leads to depolarization of adrenocortical cells, activation of voltage-dependent Ca^2+^ channels, Ca^2+^ influx, increased *CYP11B2* gene expression and aldosterone production.

### 3.3. Familial Hyperaldosteronism Type III

Choi et al. identified the genetic basis of familial hyperaldosteronism type III (FH type III) in 2011 by next-generation sequencing [6]. A gain-of-function mutation in the *KCNJ5* gene (encoding the G-protein-coupled inwardly rectifying potassium channel GIRK4) was identified in the male index case and his two daughters. The mutation results in the substitution of a threonine residue (p.Thr158Ala) located just above the selectivity filter of the channel pore which interferes with the Thr158-Pro128 hydrogen bonding [6]. Patch clamp recordings of human embryonic kidney cells expressing the mutated GIRK4 p.Thr158Ala channel showed that the mutation results in a loss of selectivity for K^+^ and permissively allows the passage of Na^+^ resulting in membrane depolarization [6]. In adrenal cells, membrane depolarization leads to the opening of voltage gated Ca^2+^ channels and Ca^2+^ influx activating the Ca^2+^ signalling pathway and aldosterone production. Expression of GIRK4 p.Thr158Ala in the human adrenocortical carcinoma cell line (HAC15) caused a marked increase in aldosterone secretion that was dependent on membrane depolarization and Na^+^ and Ca^2+^ influx [19]. Until 2017, 22 patients with FH type III were described in the literature from 12 families [20]. Notable is that over half of the patients described with FH type III (14 of 22 cases occurring in 7 of 12 families) carried mutations of the Gly151 residue (p.Gly151Glu or p.Gly151Arg) in the GlyTyrGly motif implicated in K^+^ selectivity [21].

### 3.4. Familial Hyperaldosteronism Type IV

Familial hyperaldosteronism type IV (FH type IV) is caused by gain-of-function mutations in Cav3.2, a T type Ca^2+^ channel encoded by *CACNA1H.* FH type IV was first identified in 2015 by exome sequencing of 40 unrelated subjects with early-onset hyperaldosteronism and hypertension (<10 years of age) [22]. Scholl et al. identified five subjects with the same heterozygous mutation in *CACNA1H* encoding the Ca^2+^ voltage gated channel (Ca_v_3.2) resulting in a Ca_v_3.2 p.Met1549Val substitution [16]. Comparisons of whole cell patch clamp recordings of Ca_v_3.2 p.Met1549Val and wild-type Ca_v_3.2 expressed in human embryonic kidney cells showed that the p.Met1549Val mutation causes an impairment of channel activation and inactivation. The mutant channel displayed slightly slower activation and much slower inactivation time constants compared with the wild-type channel as well as a tail current indicating that a proportion of the mutated channels remain non-inactivated. These properties would lead to an increase in Ca^2+^ influx in adrenal ZG cells and signal an increase in aldosterone production. Validation of this concept was subsequently demonstrated by the same group by expression of the Ca_v_3.2 p.Met1549Val mutation in human adrenocortical (HAC15) cells which resulted in an increase in *CYP11B2* gene transcription and aldosterone secretion relative to cells expressing the wild-type channel [23]. Following the discovery by Scholl, additional mutations in *CACNA1H* were described involving a substitution of the same Met1549 residue (Met1549Ile) or other amino acid residues (Ser196Leu, Val1951Glu and Pro2083Leu) [24].

## 4. Somatic Mutations in Aldosterone-Producing Adenomas

The most frequent genetic variation in APA is a somatic mutation of the *KCNJ5* gene [25]. First identified by Choi et al. in 2011 by exome sequencing, mutations in *KCNJ5* were identified in 8 of 22 APA resulting in GIRK p.Gly151Arg or p.Leu168Arg mutations [6]. Both mutations were demonstrated to interfere with the selectivity filter of the channel pore and result in membrane depolarization causing the opening of voltage gated Ca^2+^ channels in adrenal glomerulosa cells and Ca^2+^ influx [6]. Somatic *KCNJ5* mutations are found at a prevalence of 40–50% [26,27,28,29,30] although a higher prevalence has been reported in populations from Japan and China [31,32]. Following the description of the *KCNJ5* mutations, the application of next generation sequencing rapidly identified additional somatic mutations associated with aldosterone overproduction in sporadic PA. These include heterozygous gain-of-function mutations in Ca_v_1.3 (the α1D subunit of the L-type voltage-dependent calcium channel) encoded by *CACNA1D* [8,9] and the ion transporters, Na^+^/K^+^-ATPase (encoded by *ATP1A1*) and Ca^2+^-ATPase (encoded by *ATP2B3*) [7,8]. These mutations result in an increase in intracellular Ca^2+^ concentration thereby causing an increase in transcription of the *CYP11B2* gene that encodes aldosterone synthase. Activating mutations in exon 3 of *CTNNB1* that encodes β-catenin have been identified in APAs as well as in other adrenal tumours [33]. Despite these major advances, the mechanisms underlying the deregulated cell growth of APAs are probably not explained by somatic mutations and the GIRK4 Thr158Ala mutation does not enhance proliferation of adrenal cells in vitro [19]. Herein we discuss the transcriptome studies that have identified genes and signalling pathways that may function in the pathophysiology and pathogenesis of APA.

## 5. Gene Expression Profiling

Gene expression studies have identified genes with a potential role in the pathogenesis and pathophysiology of APAs (Table 2). Despite inter-study heterogeneity of gene expression data, which may be accounted for by the use of different reference tissues (adjacent cortex or normal adrenal tissue or, in some cases, non-functioning adrenocortical adenomas) and different diagnostic criteria [34,35,36,37,38], such studies have proven valuable in the identification of genes and signalling pathways with a potential role in the pathogenesis and pathophysiology of APAs.

Gene expression studies employing microarrays have shown a higher expression of *CYP11B2* in APA compared with normal adrenals [39,40,41] and by SAGE (serial analysis of gene expression) in an APA compared with adjacent cortical tissue [42]. However, another study of APA transcriptomes reported two distinct and opposing expression profiles for genes encoding steroidogenic enzymes with *CYP11B2* displaying increased or decreased expression levels with respect to normal adrenal tissue [43]. This apparent paradoxical decrease of the gene expressing aldosterone synthase in a tumour that overexpresses aldosterone may be accounted for by sampling areas of the normal adrenal reference tissue. In fact, these may contain aldosterone-producing cell clusters (APCC) that express high levels of *CYP11B2* with somatic *CACNA1D*, *ATP1A1* or *ATP2B3* mutations [44,45,46]. Conversely large APA with low expression of *CYP11B2* that give rise to inappropriate aldosterone production might occur. Another possibility is that non-APA nodules were used in the study due to non-selective diagnostic criteria.

**Figure 2:**
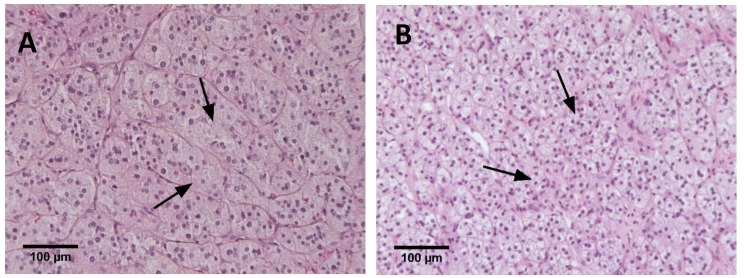
Histopathological phenotype of aldosterone-producing adenomas. Haematoxylin and eosin staining of an aldosterone-producing adenoma showing large lipid-rich cells of the ZF type (indicated with arrows) (panel **A**) or a predominance of smaller compact cells of the ZG type (indicated with arrows) (panel **B**).

Many studies have described an association of somatic APA mutations with histological phenotype. APAs carrying *KCNJ5* mutations have been widely reported to comprise predominantly large lipid-rich ZF-like cells (Figure 2) [8,47,48,49,50]. Some studies have also described a predominance of small compact ZG-like cells in APA harbouring *CACNA1D*, *ATP1A1* or *ATP2B3* mutations [8,27,49,51] and somatic APA genotype is associated with plasma steroid profiles [52]. Such genotype-phenotype associations indicate that APA genotype may influence transcriptome signatures. Histological differences between large lipid-rich ZF-like cells and small compact ZG-like cells in APA are shown in Figure 2.

No differences in the transcriptome profiles of APA with and without *KCNJ5* mutations were initially described [26]. However, later studies reported distinct expression profiles of APA with *KCNJ5* mutations compared with APA without *KCNJ5* mutations (with higher *CYP11B2* expression in the tumours with *KCNJ5* mutations) [53]. Different expression profiles were reported in APA with *ATP1A1* and *ATP2B3* mutations relative to APA with *KCNJ5* mutations (with higher *CYP11B2* expression in the tumors with *ATP1A1* and *ATP2B3* mutations) [54]. Azizan et al. [35] demonstrated marked differences in *CYP17A1* gene expression from microarrays, validated by real-time PCR, in APA with a ZF phenotype compared to those APA with a ZG phenotype [47]. If *CYP17A1* and *CYP11B2* are expressed in the same cell then cortisol can be metabolized further to produce the hybrid steroids 18-hydroxycortisol and 18-oxocortisol [55]. Higher levels of these hybrid steroids are associated with FH type I and FH type III (although not in all cases) and in patients with an APA with a *KCNJ5* mutation [12,52].

*CYP17* expression in APA has been shown to be associated with APA phenotype with marked upregulation in adenomas comprising predominantly ZF-type cells [47]. *NURR1* (*NR4A2*, encoding Nur-related factor 1) and *NGFIB* (*NR4A1*, encoding nerve growth factor IB), genes that encode transcription factors playing a key role in the regulation of *CYP11B2* gene transcription [56], are upregulated in APA. Also, genes encoding the nuclear receptor transcription factors SF-1 (*NR5A1*) and DAX1 (*NR0B1*) that are essential for adrenal development and steroidogenesis, are upregulated in APA [39]. Although low DAX1 expression in adrenocortical tumours is associated with aldosterone production [57]. A target gene of SF-1, *VSNL1* [58], is upregulated in APA and *VSNL1* in vitro overexpression in the NCI H295R cell line results in an increase in aldosterone production under both basal and angiotensin II-stimulated conditions [59].

Several genes encoding G-protein-coupled receptors are among the genes upregulated in APA, including those encoding the luteinizing hormone receptor (LH-R encoded by *LHCGR*), gonadotropin releasing hormone receptor (GnRHR encoded by *GNRHR*), serotonin receptor 4 (HTR4), melanocortin 2 receptor (MC2R), and the angiotensin II type 1 receptor (AGTR1) [60]. Overexpression of LH-R in the adrenocortical carcinoma NCI H295R cell line causes a concentration-dependent increase in *CYP11B2* expression after stimulation with luteinizing hormone [40]. Accordingly, the expression of *LH-R* and *GnRHR* in APAs has been proposed to be related to increased aldosterone production during pregnancy [61]. Therefore, the presence of activating APA *CTNNB1* mutations might contribute to an abnormal receptor activation [60].

*NEFM*, encoding the medium neurofilament protein, is highly upregulated only in APAs without *KCNJ5* mutations and is selectively expressed in the ZG and in APA comprised of predominantly ZG cells [62,63]. Dopamine regulates aldosterone production via activation of its G-protein-coupled receptor (GPCR) subtypes and silencing of *NEFM* amplified aldosterone stimulation by a DR1 (dopamine receptor subunit 1) agonist and aldosterone secretion in response to the DR1 agonist was greater in primary cultures of APAs composed of primarily ZF cells compared with cultures of APAs with ZG cells. These data indicate a role for *NEFM* in aldosterone production and cell proliferation [63].

Analysis of the methylome of APAs demonstrated hypomethylation of GPCR genes and a strong association of promoter hypomethylation of the HTR4 and PTGER1 genes with the upregulation of mRNA levels, validated by real-time PCR, was demonstrated in APAs compared with non-functioning adrenocortical adenomas [64]. Methylation of HTR4 and PTGER1 was significantly inversely correlated with their respective mRNA expression levels [64]. The most hypomethylated promoter in APA is the *PCP4* (encoding purkinje cell protein 4) promoter with demethylation associated with enhanced gene transcription [65]. *CYP11B2* was also extensively hypomethylated in APAs [64] but although hypomethylation was not associated with gene expression levels in this study it could facilitate gene transcription [64]. In contrast, Howard et al., reported hypomethylation of APAs with hypomethylation and overexpression of *CYP11B2* [64].

**Figure 3:**
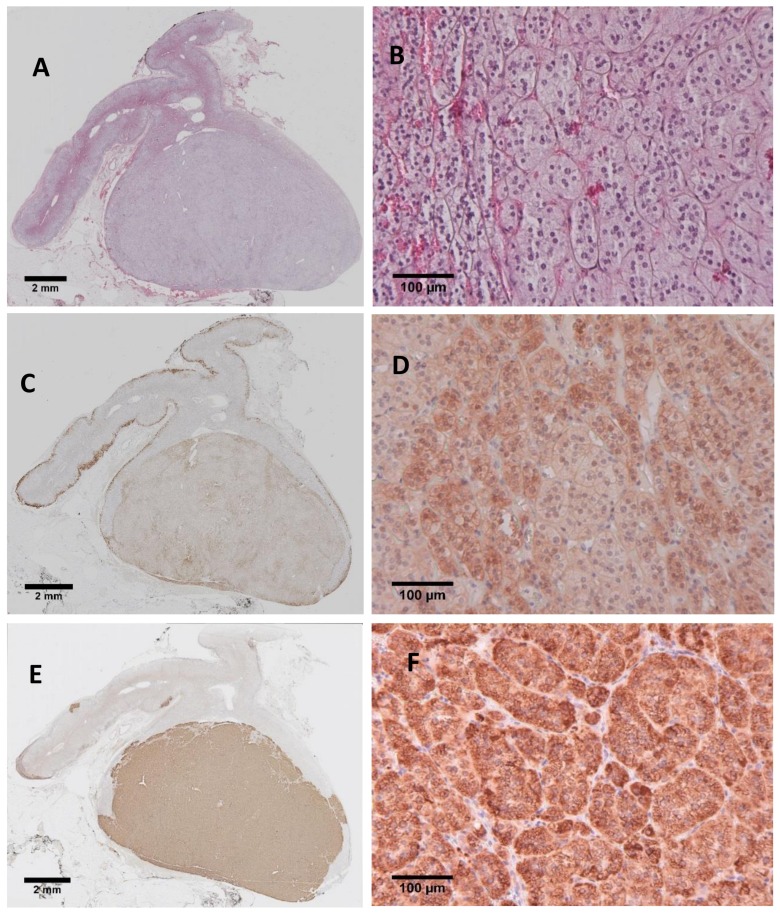
VSNL1 and CYP11B2 immunohistochemistry. An aldosterone-producing adenoma with a *KCNJ5* mutation stained with haematoxylin and eosin panels (**A**,**B**); immunostained for VSNL1, panels (**C**,**D**); and for CYP11B2 panels (**E**,**F**). The VSNL1 antibody was from Merck and the CYP11B2 was a kind gift from Prof Celso Gomez-Sanchez, University of Mississippi, Oxford, MS, USA.

Calcium is a key intracellular messenger for aldosterone production and the intracellular Ca^2+^ signaling pathway is independent of the renin–angiotensin–aldosterone system in APAs [66]. A number of genes involved in Ca^2+^ signaling or Ca^2+^ sequestration have been reported as upregulated in APAs and are described in more detail below. *VSNL1* that encodes a Ca^2+^-sensor protein and a target of the nuclear receptor SF-1 [58] was one of several upregulated genes in APAs by microarray analysis compared with normal adrenals validated by real-time PCR [41]. In NCI H295R adrenal cells, overexpression of *VSNL1* resulted in an upregulation of *CYP11B2* gene expression under both basal and angiotensin II-stimulated conditions thereby implicating a role for *VSNL1* in aldosterone production. Analysis of a larger sample set of tumours showed that *VSNL1* was overexpressed in APAs carrying a *KCNJ5* mutation compared with those APA without a *KCNJ5* mutation. A potential role for the calcium sensor in the protection of cells in an adenoma via Ca^2+^-related anti-apoptotic cell death mechanisms was hypothesized [59]. The expression of the VSNL1 protein in an APA (carrying a *KCNJ5* mutation) that displays strong CYP11B2 immunostaining is shown in Figure 3.

The *CALN1* gene, that encodes the Ca^2+^ binding protein calneuron 1, has been reported as upregulated in APA in two transcriptome studies [41,67]. CALN1 was shown to potentiate aldosterone production and silencing *CALN1* led to a decrease in Ca^2+^ storage in the endoplasmic reticulum and abrogated angiotensin II-mediated aldosterone secretion in an adrenocortical carcinoma cell line [41].

*CALM2* encoding calmodulin 2 is a Ca^2+^-binding protein expressed in a wide-range of tissues involved in signalling, cell cycle progression and proliferation. *CALM2* was highly upregulated in a transcriptome comparison of APAs with the adjacent ZG [42]. The increased expression of *PCP4* in APA cells is likely to play a role in APA pathophysiology because PCP4 modulates Ca^2+^-binding by calmodulin and activates the calcium-calmodulin cascade leading to an increased expression of *CYP11B2* [68].

*GSTA1* (encoding glutathione-*S*-transferase, an enzyme that protects cells from reactive oxygen species, ROS) gene expression is inversely correlated with the level of aldosterone production in APAs with a *KCNJ5* mutation and appears to regulate aldosterone secretion via ROS and Ca^2+^ signalling [69]. *GSTA1* overexpression suppressed aldosterone biosynthesis, while silencing of *GSTA1* increased aldosterone production through increasing ROS, superoxide, H2O2 levels, Ca2+ influx and the expression of *CAMK1* (encoding Ca^2+^/calmodulin dependent protein kinase 1) and the transcription factors *NR4A1* (also called *NGFIB*) and *NR4A2* that regulate *CYP11B2* gene expression [69].

The epidermal growth factor-like teratocarcinoma-derived growth factor-1 gene (*TDGF1*) was identified as the most highly expressed gene in APAs compared with normal adrenals by microarray analysis [41]. *TDGF1* was also identified as upregulated in an APA relative to the paired adjacent cortex by serial analysis of gene expression [41]. Overexpression of *TDGF1* in NCI H295R adrenal cells activated the PI3K-Akt signalling pathway and led to an increase in aldosterone production, indicating a potential role in APA pathophysiology [41]. The activation of PI3K/Akt mTOR signalling, a pathway with a known role in cell proliferation, was also reported in patients with PA [41].

Wnt plays a key role in the development of the adrenal cortex and the dysregulation of this signalling pathway is associated with tumorigenesis [70]. The Wnt/β-catenin pathway is constitutively activated in around 70% of APAs [71] with the decreased expression on the Wnt inhibitor *SFRP2* (encoding secreted frizzled related protein 2) likely playing a role in the deregulated Wnt/β-catenin signalling [72]. *SFRP2* was also four-fold down-regulated in APAs compared with normal adrenals in an oligonucleotide microarray [72]. Mice with an ablation of *Sfrp2* display enhanced aldosterone production [72]. β-catenin appears to mediate aldosterone production by increasing the transcription of several genes including *AT_1_R*, *CYP21* and *CYP11B2* as well as upregulating expression of transcription factors NURR1 (*NR4A2*) and NUR77 (*NR4A1*) [72].

*NPNT* (nephronectin), a secreted matrix protein, was most highly expressed in APAs with a ZG phenotype with *CTNNB1* mutations. Thereby it may represent a potential biomarker to recognize a subtype of APAs and indicates a further mechanism by which the Wnt/β-catenin signalling pathway may upregulate aldosterone production [72]. These studies show that aberrant Wnt/β-catenin pathway activation is associated with APA development and suggests that the Wnt/β-catenin signalling mediates aldosterone production at multiple levels [71].

## 6. Single Nucleotide Polymorphisms

Single nucleotide polymorphisms (SNPs) are the most frequent genetic variation in the human genome. A rare nonsynonymous SNP (rs7102584) resulting in a GIRK4.p.Glu282Gln substitution was identified in 12 of 251 patients (5%) with sporadic PA (9 with bilateral and 3 with unilateral PA) compared with a prevalence of 2% in the 1000 genomes cohort [73]. Five common SNPs of the *KCNJ5* gene (rs6590357, rs4937391, rs3740835, rs2604204, and rs11221497) were found in patients with sporadic PA and essential hypertension and a significant association of the rs2604204 variant with sporadic PA in Chinese males was found indicating a potential role for this polymorphism in the pathogenesis of sporadic PA in this specific subgroup of patients [74].

## 7. Influence of Genetics and Genomics on Surgical Outcome

High blood pressure may persist after adrenalectomy due to contributory factors other than PA and the surgical cure rate of patients with APAs varies widely. Comparison of the transcriptomes of APAs with normal adrenals identified two subgroups of APAs based on their expression profiles (low *versus* high mRNA levels) of genes encoding steroidogenic enzymes. APAs with a low level of *CYP11B2* gene transcription are associated with a longer known duration of hypertension and a lower rate of long-term cure [43].

Microarray analysis identified differentially expressed genes in a comparison of the transcriptomes of APAs from patients with persistent hypertension after adrenalectomy with that of patients with APA who were cured by surgery [75]. The differentially expressed genes were associated with five different pathways that included lipid metabolism and cell differentiation and indicate the possibility of using genomic approaches to identify drug targets and prognostic markers [75].

A number of studies have investigated the effect of *KCNJ5* mutational status as a marker for surgical outcome. In a prospective study by the TAIPAI (Taiwan Primary Aldosteronsim Investigation study group) of 108 patients that were divided into *KCNJ5* mutated and non-mutated groups, patients with an APA carrying a *KCNJ5* mutation aged between 37 and 60 years may have an advantage in blood pressure response to surgery but mutation status is not associated with an improvement in arterial stiffness [76].

Cardiovascular complications before and after unilateral adrenalectomy in patients harboring APAs with and without *KCNJ5* gene mutations were evaluated in a Japanese population. The *KCNJ5*-mutated group displayed a significant improvement in left ventricular mass index which was independently associated with the presence of APA *KCNJ5* mutations whereas the group without *KCNJ5* mutations had no such improvement [77]. A higher left ventricular mass index and plasma aldosterone concentration in patients with APA *KCNJ5* mutations relative to those without *KCNJ5* mutations has also been reported [35]. Despite the increased cardiac damage, patients with *KCNJ5* mutations exhibited a decrease of blood pressure and plasma aldosterone concentrations and a regression of left ventricular mass index similar to the *KCNJ5* wild type group after adrenalectomy [35]. Another study reported an association of APA *KCNJ5* mutations with lower blood pressure and the higher likelihood of cure of PA by adrenalectomy relative to patients with APA without *KCNJ5* mutations [36].

## 8. Conclusions and Perspectives

Major discoveries have been made in the field of PA research over the last few years mainly due to the application of next-generation sequencing methods. Four familial forms of hyperaldosteronism are now recognized with the genetic basis of three of these uncovered by exome sequencing. Somatic mutations have been identified in ion channels and transporters that alter intracellular ion homeostasis and drive the constitutive aldosterone production in over half of aldosterone-producing adenomas. Differential gene expression studies have further highlighted key signalling pathways and molecular mechanisms that may drive cell proliferation and aldosterone overproduction in aldosterone-producing adenomas. Transcriptome analysis methods may have a future application in the identification of prognostic markers to identify post-operative cardiovascular events.

## Figures and Tables

**Table 1:** Familial forms of hyperaldosteronism.

Subtype of Primary Aldosteronism	Genetic Variant	Encoded Protein	Brief Description
FH Type I	*CYP11B1/CYP11B2* hybrid gene	*CYP11B2*	Ectopic expression in ZF; regulated by ACTH
FH Type II	*CLCN2* mutations	CIC-2	Chloride voltage-gated channel 2
FH Type III	*KCNJ5* mutations	GIRK4	Potassium Voltage-Gated Channel Subfamily J Member 5
FH Type IV	*CACNA*1H mutations	Cav3.2	Calcium Voltage-Gated Channel Subunit α1H

**Table 2:** Differentially expressed genes in aldosterone-producing adenomas and their reference tissues used in transcriptome studies.

Gene	Encoded Protein and Description	Reference Tissue	Ref.
**Upregulated Genes**			
*CYP11B2*	Aldosterone synthase- steroid hydroxylase cytochrome P450 enzyme with 11β-hydroxylase, 18-hydroxylase and 18-oxidase activities	AAC; NLA	[34,39,40,41]
**Calcium Signaling**			
*VSNL1*	Visinin-like 1, calcium sensor protein of visinin/recoverin subfamily	NLA	[59]
*CALN1*	Calneuron 1, calcium-binding protein with high similarity to calmodulin family	NLA	[41,67]
*CALM2*	Calmodulin 2, calcium-binding protein of calmodulin family.	Adjacent ZG	[42]
*PCP4*	Purkinje cell protein 4, regulates calmodulin activity by modulating calcium binding by calmodulin	NFA	[68]
**Nuclear receptor Transcription Factors**			
*NR4A1*	Nuclear receptor subfamily 4 group A member 1; steroid-thyroid hormone-retinoid receptor superfamily.	WT-*KCNJ5*-APAs	[56]
*NR4A2*	Nuclear receptor subfamily 4 group A member 2; steroid-thyroid hormone-retinoid receptor superfamily.	WT-*KCNJ5*-APAs	[56]
*NR5A1*	Nuclear receptor subfamily 5 group A member 1 (SF1); transcriptional activator of sex determination.	AAC	[39]
*NR0B1*	Nuclear receptor subfamily 0 group B member 1 (DAX1); functions in proper formation of adult adrenal gland formation.	AAC	[39]
**G-protein-coupled Receptors**			
*LHCGR*	Luteinizing hormone/choriogonadotropin receptor	NLA	[60]
*GNRHR*	Gonadotropin releasing hormone receptor	NLA	[60]
*HTR4*	5-hydroxytryptamine receptor 4	NLA; NFA	[60,64]
*PTGER1*	Prostaglandin E receptor 1	NFA	[64]
*MC2R*	Melanocortin 2 receptor	NLA	[60]
*AGTR1*	Angiotensin II receptor type I	NLA	[60]
Others			
*NEFM*	Medium neurofilament protein- biomarker of neuronal damage	*KCNJ5*-mut APAs; ZF-like APAs	[62,63]
*TDGF1*	Teratocarcinoma-derived growth factor 1- signaling protein that functions in development and tumor growth	NLA	[41]
*NPNT*	Nephronectin, a secreted matrix protein	NLA	[72]
**Downregulated Genes**			
*GSTA1*	Glutathione S-transferase alpha 1- member of a family of enzymes that protect cells from reactive oxygen species	WT-*KCNJ5*-APAs; NLA	[69]
*SFPR2*	Secreted frizzled related protein 2- agonist of Wnt signaling	NLA	[72]

AAC: adjacent adrenal cortex; APAs: aldosterone-producing adenomas; *KCNJ5*-mut APAs: APAs with *KCNJ5* mutations; NFA: non-functioning adrenocortical adenomas; NLA: normal adrenals; WT-*KCNJ5* APAs: APAs with wild type *KCNJ5* gene; ZF: *zona fasciculata*; ZG: *zona glomerulosa*.
